# Changes, interactions, and relationships: an examination of language learning motivational system of Chinese multilingual learners

**DOI:** 10.3389/fpsyg.2026.1864018

**Published:** 2026-08-05

**Authors:** Qing Xu, Jinghui Si

**Affiliations:** School of Foreign Languages, Shenzhen Technology University, Shenzhen, China

**Keywords:** changes, emotion, motivational system, multilingualism, relationships

## Abstract

Motivation is of great significance in understanding multilingual development. This study closely examined the motivational system of Chinese university students in the process of multilingual learning. A retrospective questionnaire survey was conducted with 97 English majors, among whom 17 were invited to participate in semi-structured interviews. The findings revealed that Japanese temporarily surpassed English in motivational intensity. This was attributed to a sense of novelty arising from learning a new additional language in tandem with a feeling of fatigue due to a perceived discrepancy between ‘should-be L2 learner’ and ‘actual L2 learner’. Later, English learning motivation reasserted its dominance in an intricate interplay among identity, value, and motivation. This is indicative of an identity-motivation nexus and a structurally conditioned motivational system. Moreover, a tension between subjective metacognitive framing and the lived experiences was found. While learners described their English and Japanese motivational systems as separate and in parallel, their accounts simultaneously revealed instances of motivational interactions. This points to a divergence between perceived and enacted motivational dynamics. This article concludes with some pedagogical implications for language learning and teaching.

## Introduction

Learning additional languages is a developmental process, and achieving proficiency requires persistent motivation (Dörnyei and Ryan, 2015). Research on language learning motivation has traditionally developed within the framework of second language acquisition (SLA), with a predominant focus on learners’ engagement with a single target language (Ushioda, 2016). Early motivational models emerged largely from bilingual contexts and were primarily concerned with explaining success, persistence, and achievement in second language (L2) learning. As English has consolidated its position as a global lingua franca, this focus has become increasingly concentrated on English, with a substantial proportion of empirical studies examining motivation for learning English as a foreign language (EFL). Consequently, motivation for learning additional languages other than English has remained comparatively underexplored.

In recent years, SLA has undergone a “multilingual turn” (May, 2019; Ortega, 2019). More recent research has emphasized that languages have soft boundaries and language learning is diverse (Cenoz and Gorter, 2022; Jessner, 2008). While an individual’s whole linguistic repertoire is unified, fluidic and dynamic, a well-established distinction between a ‘first’ or native language, a ‘second language’ (L2) and a third language (L3) still has significant research, educational, and political meanings (Jessner et al., 2025). Hammarberg (2010) defined an L3 as “a non-native language which is currently being used or acquired in a situation where the person already has the knowledge of one or more L2s in addition to one or more L1s.” Thus, an L3 learner brings prior knowledge in at least one L1 and one L2, while the L3 is in the process of being added to the learner’s linguistic repertoire. It has been argued that multilingual learners do not possess isolated motivational systems for each language, but rather experience multiple, coexisting language motivations that may influence one another (Bui et al., 2018). However, the literature on motivation thus far has focused almost exclusively on psychological factors, motivational currents, ideal self, and self-regulated learning on L2. Less is known about the dynamic nature of L3 motivation. Even less is known about the interaction between L2 and L3 motivation. As a consequence, two empirical gaps persist. First, the extant literature has predominantly examined the influence of L2 on L3 motivation, while the reverse influence, or how engagement with an L3 may be perceived to affect motivation for L2 English, has received comparatively little attention. Second, there is a limited understanding of how multilingual learners themselves *interpret, narrate, and rationalize* changes in their motivation across languages in retrospection. That is, while prior research has documented patterns of motivational currents, fewer studies have focused on learners’ retrospective meaning-making processes. In other words, how they explain perceived motivational changes, identify turning points, and articulate cross-language influences within their own learning histories.

Addressing these gaps, the present study investigates Chinese English majors who study Japanese as a third language. According to a Japan Foundation survey, as of 2024, China has approximately 1.019 million learners of Japanese, which renders the country the largest population of Japanese learners worldwide. Among them, 477,869 learners are enrolled in higher education institutions, accounting for approximately 46.88% of the total (Japan Foundation, 2024). Given the substantial proportion of university-level learners, an investigation of the motivational systems of English and Japanese multilingual learners in higher education holds significant pedagogical relevance.

Rather than attempting to model motivational dynamics longitudinally, the study adopts a retrospective and interpretive approach. It examines learners’ self-reconstructed motivational patterns and their perceived cross-language influences. Drawing on Complex Dynamic Systems (CDST) as a conceptual framework, the study explores how learners describe periods of relative motivational stability, perceived disruptions, and phase-like changes in their engagement with L2 English and L3 Japanese. By foregrounding learners’ subjective accounts, this study aims to contribute to a more nuanced understanding of multilingual motivation as a lived, interpreted experience shaped by institutional demands, future-oriented goals, and personal interests, within the broader context of higher education.

### L2 motivational system and the multilingual self

In early research on second language learning motivation, language learning motivation was categorized into instrumental motivation and integrative motivation (Gardner and Lambert, 1972). The notion of integrative orientation has, however, been largely challenged with an accumulating body of research on World Englishes and English as a lingua franca (Fang, 2025). In his seminal work on the dynamic nature of motivation, Dörnyei (2005) proposed the L2 motivational self-system, which consists of the ideal L2 self, the ought-to L2 self, and the L2 learning experience. The ideal L2 self represents an individual’s envisioned identity as a competent user of a second language, along with the desire to reduce the discrepancy between the current self and ideal self. The ought-to L2 self is shaped by external pressures and expectations that motivate learners to reach a certain level of language proficiency, failure of which may result in unfavorable social outcomes (Thu and Lee, 2025). The L2 learning experience concerns learners’ context-specific motives that arise from their learning environment and experiences (Dörnyei, 2009). Dörnyei (2005) posited that motivation arises from the interplay between the ideal and ought-to self. Research has further substantiated that although both the ideal and ought-to L2 selves provide the initial impetus for learning a second language, it is the quality of the L2 learning experience that ultimately determines whether this motivation is maintained or diminished over time.

The L2 motivational self-system has inspired a bulk of research on the relationships between motivation and learning outcomes. The research, as Ortega (2013) argued, has a deep-rooted monolingual bias and marks a disproportionate focus on English. As research on motivation to learn languages other than English in multilingual settings attracts scholarly attention, the established self-system is believed to be adequate in understanding the interactions of system-level motivational phenomena. Building on this, Henry (2017) proposed the concept of the ideal multilingual self, as one of the multilingual self-guides emerging from interactions between different L2 motivational system. According to Henry and Thorsen (2018), the multilingual ideal self encompasses the holistic multilingual experiences while reflect deeper personal, emotional and cultural values attached to being or becoming multilingual, and thereby influence motivation. Subsequent studies have substantiated that motivation in multilingual learning was constructed through the interplay between ideal multilingual self and current learning experiences, during which they interacted with their situated contexts and negotiated the value of learning a LOTE (Wang and Fisher, 2023). As a consequence, the multilingual posture that emerged from the self-motivated learners may overcome the monolingual bias while enable to construct the idea of an imagined global community (Zheng et al., 2020). This points to the need to for a deeper understanding of the complex motivational interactions between global English and LOTEs in multilingual learning.

### The relationship between L2 and L3 motivation

Numerous studies have explored the relationship between L2 English and LOTEs, In many Western and European contexts, studies have suggested that English, due to its global status and instrumental value, may exert a suppressive effect on L3 motivation. The process of learning less commonly used languages thus takes place in the shadow of English (Dörnyei and Al-Hoorie, 2017). Moreover, different languages are associated with distinct ideal selves, from which potential competition may emerge (Dörnyei and Chan, 2013). In other words, languages ‘compete’ for limited linguistic and cognitive resources, and the apparent winner tends to be the English language.

However, findings from Asian contexts complicate this narrative. Several studies report that English does not necessarily undermine motivation for learning additional languages. Instead, learners may view multilingual competence as an asset, perceiving English as necessary but insufficient for personal development, academic advancement, or career competitiveness (Wang and Zheng, 2021). In such cases, L3 learning may be motivated by cultural interest, identity construction, or differentiation in increasingly competitive educational and employment markets. These studies suggest that the relationship between L2 English and L3 motivation is not uniformly competitive, but context-dependent and learner-specific (Wang and Liu, 2020).

Among studies investigating the reciprocal impact of L3 on L2, research set in the Hong Kong context reveals that L3 Japanese exerts predominantly dampening effects on L2 English, manifested in the phenomena of limited cognitive resource prioritization, cross-linguistic interference (including deteriorated oral proficiency and weakened L2 English usage habits), and reformulate ideal L2 self, lower proficiency expectation for English (Bui and Teng, 2021).

Taken together, much of the existing literature has focused primarily on the *directional influence of L2 English on L3 motivation*. Fewer studies have explored how engagement with an L3 may be perceived to influence motivation for English, particularly in contexts where English occupies a dominant institutional role. Moreover, when bidirectional relationships are discussed, they are often inferred from quantitative correlations. Few studies have touched upon learners’ own explanations of how and why such influences occur.

### A holistic and dynamic view of multilingualism and motivation

This study takes a holistic and dynamic view of multilingualism and motivation. As emphasized by Duff (2017), “just as linguistic knowledge itself is not strictly compartmentalized or modularized in the mind,” motivational selves are to be viewed as interrelated, dynamic, complex systems operating—and emerging—across multiple languages, not as sperate systems” (p. 599). Therefore, *multilingual* in this study is not recognized as discrete and bounded systems that can be separated and counted (Li, 2018), and the names languages are only used to for their relevance from an emic perspective.

Given the complexity of multilingualism and motivation, a dynamic perspective is employed in research on multlingualism and on motivation, respectively. For instance, Herdina and Jessner (2002) developed what they call as the Dynamic Model of Multilingualism (DMM), which intends to provide a conceptual toolkit for analysing the psycholinguistic behaviors exhibited by multilingual individuals. Although being critiqued as not successfully shown why and how multilingualism is complex in a way that makes it special, the model is deemed stimulating and valuable in challenging the monolingual bias (Beisbart, 2021).

In line with the fluid, boundary-transcending theorisation of multilingualism, L2 motivation research underpinned by Complex Dynamic Systems Theory (CDST) advocates a holistic and dynamic view of motivation. This challenges the static and binary understanding of motivation (Zheng et al., 2020). The holistic and dynamic paradigm conceptualizes motivation as an open, self-organizing and situated ecological system, in which motivational orientations, identities, and contextual factors interact and negotiate in a dynamic way. In an attempt to reconceptualize L2 motivation from a CDST perspective, Papi and Hiver (2020) carried out a retrospective narrative study on English learning motivation of six Iranian graduates in the United States. The findings confirm that motivation is a relational system where time, context, and the interdependence of cognitive, affective, and social components are central to understanding its development.

Among the limited studies that empirically investigate the self-guided motivational systems in multilingual learning from a dynamic perspective, Thompson (2017) explored the motivational profiles of two LOTE learners of Chinese and Arabic. The findings uncovered the potential synergistic relationship between learners’s ought-to selves and anti-ought-to selves, alongside the inherent nonlinear complexity of each individual’s dynamic linguistic system. This study further demonstrates how contextual elements interact with motivational systems throughout the language learning process. Similarly, Zheng et al. (2020) tracked the evolution of Chinese learners’ multilingual motivation through a longitudinal approach. The authors argued that multiple facets of motivation in multilingual learning do not operate in isolation, but rather coexist and interact through interconnected relationships within the holistic, dynamic, and relational system that constitutes the multilingual self.

Following the line of investigating motivation in multilingual learning from a dynamic perspective, this study employs CTSD to conceptualize and investigate motivation as a dynamic, interconnected, and context-dependent system (Larsen-Freeman, 2017). It seeks to explore how Chinese English majors retrospectively reconstruct and interpret the motivational dynamics between their L2 English and L3 Japanese learning experiences. Of particular interest to this study was how learners perceive motivational changes, cross-language interactions, and what are the meaning-making processes through which learners narrate and rationalize these changes over time.

In doing so, it aims to address two significant gaps in the extant literature. First, while prior research has predominantly examined how motivation for learning English as an L2 affects motivation for learning additional languages (LOTEs), how engagement with an L3 may feed back into and reshape L2 motivation, has received little attention. In other words, far less is unknown about the relational dimension of the multilingual motivation system. Second, although previous studies have documented patterns of motivational change, fewer have focused on the intensity changes of motivational subsystems and how learners themselves interpret, narrate, and rationalize these changes in retrospect, particularly in terms of how they explain perceived motivational changes, identify key incidents, and articulate cross-language influences from their own learning experiences.

## The present study

Drawing on a holistic and dynamic view of motivation, this study aims to explore the temporal evolution and co-adaptive interactions between the motivational systems of Chinese multilingual learners of English (L2) and Japanese (L3). Specifically, the study addresses the following research questions:

1. How do learners perceive and interpret the changes in the motivational system?
2. In what ways do learners perceive and interpret the relationships between the L2 and L3 motivational subsystems?

### Research design

Given the complex and dynamic nature of the motivational system, the research design drew on the retrodictive qualitative modeling (RQM) developed by Dörnyei (2014). RQM is believed to be able to maximize the potential to understand dynamic moves in a systematic manner regardless of idiosyncratic differences (Gu, 2023). By tracking back the interpretations of changes of motivational intensity as well as the reasons why the system has ended up with a particular prototype, the study pinpoints the main factors that shape learners’ meaning-making processes of changes, interactions, and relationships between motivational subsystems.

In this research, the first stage of RQM involved exploring the changes of motivational intensity of multilingual learners and mapping out the prototypical phase-based motivational changes. This was carried out through administering a questionnaire survey. The second step started with purposively sampling learners that are representative of the established prototypes. The identified students were then invited to take part in semi-structured interviews to elicit informative perceptions and experiences. The third step centerd on self-interpretation of motivational changes and factors that shape the changes. Once the main factors were identified, efforts were put to pinpoint how these factors interacted with each other in producing the motivational trajectories.

### Participants

The participants in this study were English majors who had learnt Japanese as an L3. The first author contacted faculty members of the School of Foreign Language Studies and recruited participants using a snowball sampling strategy. Participants were informed of the purpose of the study, the voluntary nature of their involvement, and their right to withdraw at any stage. Written informed consent was obtained from all participants. In total, 97 English majors were recruited. All the respondents had been learning English for 10–15 years, and Japanese since Semester 3. Among all the questionnaire respondents, 17 participated the semi-structured interviews (See Table 1 for demographic information).

**Table 1:** Demographic information of interviewees.

Participant	Gender	Year	Major	Japanese learning experience
N1	M	3rd	English	1 Y 2 M
N2	M	3rd	English	1 Y 2 M
N3	F	3rd	Business English	1 Y 2 M
N4	F	3rd	English	1 Y 2 M
N5	F	3rd	English	1 Y 8M
N6	M	3rd	English	1 Y 2 M
N7	F	3rd	English	1 Y 8M
N8	F	3rd	English	1 Y 8 M
N9	F	4th	English	2 Y
N10	F	3rd	English	1 Y 2 M
N11	M	2nd	English	10 M
N12	F	2nd	English	2 M
N13	F	2nd	English	2 M
N14	F	2nd	English	2 M
N15	M	2nd	English	2 M
N16	F	2nd	English	2 M
N17	F	2nd	English	2 M

### Instruments

Drawing on Dörnyei (2014), and Miura (2010), the study employed a retrospective questionnaire survey to investigate the complexity of the motivational system. The questionnaire consisted of three parts. The first part is designed to obtain personal information, including gender, mother tongue, and foreign language learning experience. The second component consisted of a retrospective task on motivational intensity. Participants were asked to recall and rate their motivation for learning English as an L2 and Japanese as an L3 for each semester on a 10-point scale, and to provide explanations for any fluctuations they reported over time. The third section invites participants to discuss their views on the interactions between language learning motivational systems.

Following the questionnaire survey, retrospective interviews were conducted in Mandarin Chinese to gain more insights into the motivational changes and participants’ perceptions. The interviews were guided by a list of pre-designed questions that correspond to the survey items (See Appendix A).

### Data collection

To capture the dynamic nature of the motivational system, we opted for selecting two time points to elicit the survey data. Time 1 was from June to July 2025 when the second-grade cohort (28 students) had studied Japanese for two semesters and the third-grade cohort (44 students) had studied for four semesters. Time 2 was around May 2026 and 25 more second-grade students were invited to participate the questionnaire survey. During the time of the study, the first author was the Japanese instructor for the participants. For data collection, she administered the questionnaire survey in person in one of the class periods.

The following retrospective interviews last approximately 30–60 min. During the interviews, the interviewer adopted a receptive stance and attentively listened to the participants accounts. All the interviews were recorded and transcribed verbatim for data analysis.

### Data analysis

Descriptive statistical analyses of the questionnaire data were conducted using SPSS 24, with which means, standard deviations, and distributional patterns were obtained to examine overall motivational intensity for English as L2 and Japanese as L3 across semesters. Paired-samples t-tests were also carried out to compare English and Japanese motivational intensity across semesters.

**Figure 1:**
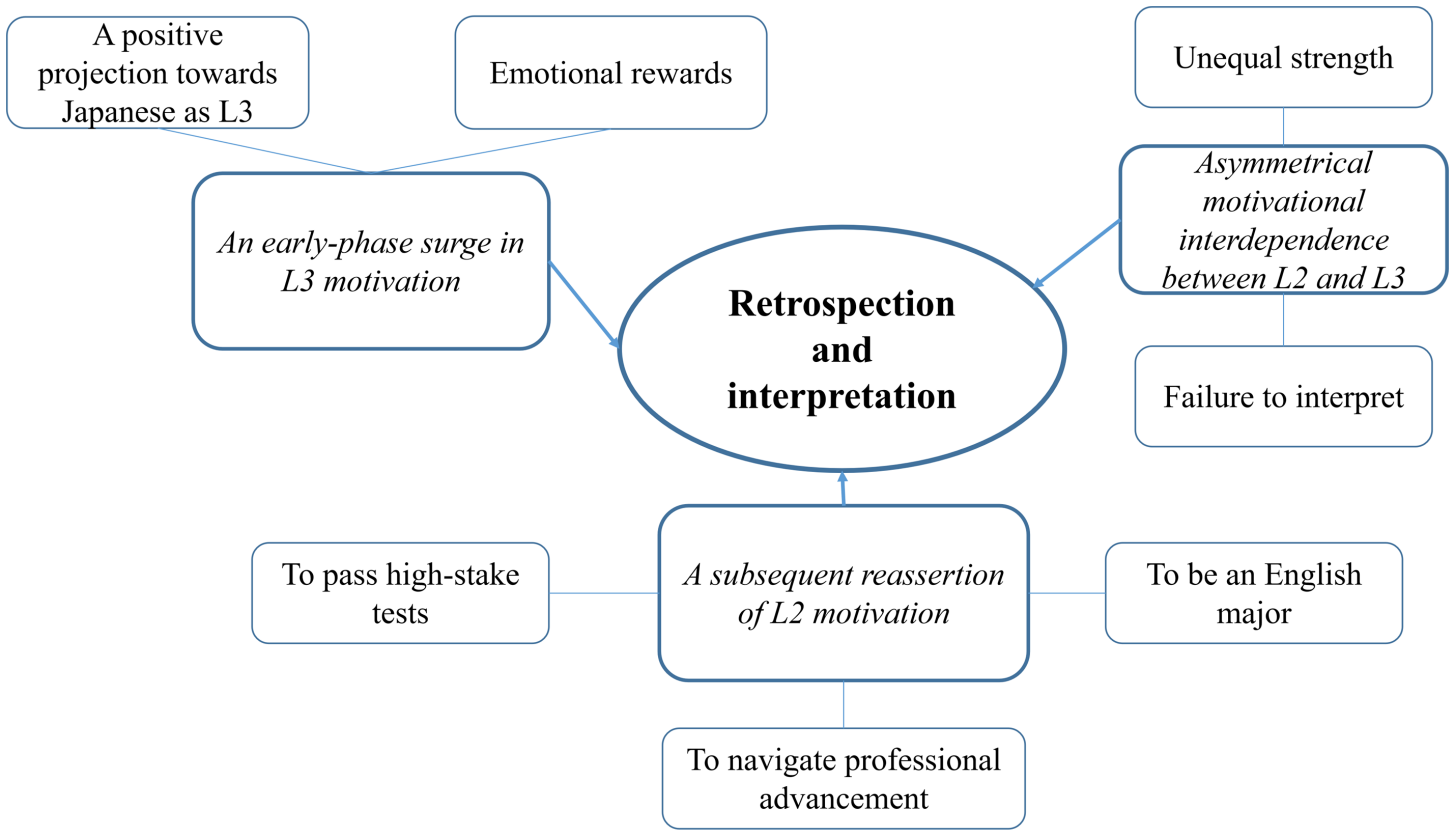
The thematic map.

Then, thematic analysis was employed to analyze the interview data. Initial open coding was conducted to capture meaningful units of data. These codes were subsequently grouped into higher-order categories through constant comparison. Three overarching themes were then identified, namely, *L3 as a motivational reorientation in the early phase*, *the reassertion of L2 motivation as long-term capital*, and *asymmetrical motivational interdependence between L2 and L3*. To interpret the signature dynamics, the relationship among the codes and themes were mapped out (See Figure 1). To enhance trustworthiness, the analysis was conducted by two researchers and carefully revisited multiple times to enhance trustworthiness.

## Findings

### Tracking the changes

**Figure 2:**
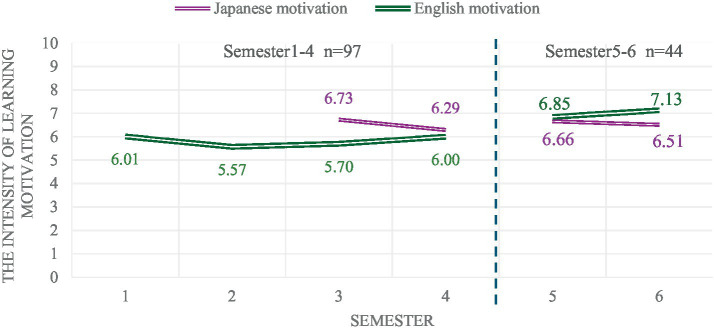
Mapping the changes.

The motivational change of learning English as L2 and Japanese as L3 was presented in Table 2 and Figure 2. Overall, English learning motivation demonstrates a generally upward trajectory over time, whereas Japanese learning motivation fluctuates moderately and exhibits a slight decline toward the end of the observed period.

**Table 2:** Means and SDs of English and Japanese learning motivational intensity.

Semester	1 (*n* = 97)		2 (*n* = 97)		3 (*n* = 97)		4 (*n* = 97)		5 (*n* = 44)		6 (*n* = 44)	
	Mean	SD	Mean	SD	Mean	SD	Mean	SD	Mean	SD	Mean	SD
English	6.01	2.27	5.57	2.15	5.70	1.76	6.00	2.12	6.85	1.53	7.13	1.73
Japanese					6.73	1.90	6.29	2.00	6.66	1.82	6.51	2.08

As shown in Figure 1, English motivation begins at a relatively high level (6.01), followed by a slight decrease in Semester 2 (5.57). It then gradually increases from Semester 3 (5.70) to Semester 4 (6.00), and continues to rise more markedly in Semesters 5 (6.85) and 6 (7.13), reaching its peak at the end of the six-semester period. This pattern suggests a steady strengthening of motivation to learn English over time, particularly in the latter half of the study.

By contrast, Japanese motivation was reported at a comparatively high level (6.73). It declines in Semester 4 (6.29), recovers slightly in Semester 5 (6.66), and then decreases again in Semester 6 (6.51). Although the fluctuations are not dramatic, the overall trend indicates a modest downward tendency after the initial measurement, with motivation levels stabilizing at a moderate level.

A paired sample t-test was conducted to compare the motivational intensity of English and Japanese learning across Semesters 3–6. There was a statistically significant difference in motivational intensity between English and Japanese learning in Semester 3, with Japanese motivation substantially higher than English motivation. However, no statistically significant differences were observed from Semester 4 to Semester 6 (Table 3).

**Table 3:** Paired samples T-test of English and Japanese learning motivation (Semesters 3–6).

English-Japanese motivation	Paired differences				t	df	Sig. (2-tailed)
	*N*	Mean	SD	Std. error mean			
Semester 3	97	−1.03	2.10	0.213	−4.81	96	0.000
Semester 4	97	−0.289	2.44	0.248	−1.16	96	0.247
Semester 5	44	0.193	1.96	0.296	0.65	43	0.517
Semester 6	44	0.614	2.55	0.385	1.60	43	0.118

### Understanding interactions and relationships

#### An early-phase surge in L3 motivation

Reflecting on the question of why Japanese as L3 elicits a multitude of positive projections, including a range of self-perceived shorter L1-L3 linguistic distance, cultural interests, and higher instrumentality in postgraduate examination, as well as expressions of the influence of social milieu. For instance:

N4: I might want to prepare for the postgraduate entrance exam, where Japanese is a common option.

N6: Japanese is kind of similar to Chinese… so I think it’s easier to learn compared with other languages.

N8: I’ve watched quite a lot of animation, so I’ve been exposed to a lot of Japanese. Compared to other languages, I’m more familiar with it.

N17: And I think Japanese pronunciation is kind of cute. It sounds really adorable.

These positive projections are related to a self-construction of Japanese as a particular kind of ‘ought-to’ language, primarily based on cross-linguistic comparison and potential cultural contact. In their perceptions, Japanese as L3 ought to be an easy-to-learn, interesting, and exam-friendly language. These perceptions lead to a novelty-driven motivation surge in relation to learning Japanese as L3. This partly explains the relatively high intensity of L3 motivation when Japanese is formally taught and learnt.

Another reason for the high L3 motivation is that the timing of starting to learn Japanese coincided with a strong sense of fatigue in relation to English language learning due to perceived stagnation or underperformance. Against this backdrop, Japanese is labeled a satisfactory outlet for alleviating such a sense of fatigue, both emotionally and psychologically. As pointed out by the following interviewee:

N13: When L3, which is completely different from Latin-based languages, appears, it gives my brain a break and provides a reprieve from all the troubles and issues associated with learning English.

According to the interviewee, the contrast between the freshness of beginning to learn Japanese as L3 and the cumulative weariness from long-term English learning makes the disparity in motivational intensity peak in the third semester. Importantly, motivation for learning English, similar to that for Japanese, starts off strong and then declines. What differs is that Japanese motivation is shaped by conceptualizing L3 as an should-be language (easy-to-learn, interesting, and exam-friendly), whereas English motivation stems from a perceived should-be L2 learner. Interviewees consistently mentioned that they had done well in English during high school and performed satisfactorily on the Gaokao English exam, which led them to choose English as their major. They reckoned that they ought to be qualified English learners. However, after entering university, their performance was not as expected, falling short of their should-be L2 learner. For example:

N4: My strong performance in high school English led me to major in English… However, I felt I did not do well, not as I hoped… I have been unable to achieve native-like proficiency.

As can be seen from the above excerpt, the Standard English Ideology seems to challenge the preconceived should-be L2 learner. As the interviewee N4 expressed, the goal of achieving ‘native-like proficiency’ feels out of reach, and subsequently, the preconceived notion of being a competent L2 learner is questioned. When learners like N4, who excelled in high school, transition to a university environment where the gap between their performance and this ‘standard’ becomes more apparent, there is a growing awareness of the vast distance between their current L2 learner and the experience-charged should-be L2 learner. The internalization of this gap seems to inhibit L2 motivation in the transitioning stage from high school to university.

Interestingly, although learning an additional language that brings a strong sense of novelty and pleasure may enhance language learning motivation at the initial stage, it does not necessarily facilitate the construction of a multilingual self. Indeed, learners may not even perceive the additional language as a generic component of their whole linguistic repertoire. For example:

Interviewer: How strong would you say your multilingual identity is, if you were to rate it on a scale of 1 to 10?

N 16: I would give it 2.5.

Interviewer: What does this multilingual identity in your mind look like? Could you first tell me roughly how many languages you imagine it includes, and how it is structured?

N16: I might lack a bit of imagination… I am quite competitive, so I do want to have a relatively high level of proficiency…. Only additional languages with high proficiency fulfill the multilingual identity.

When asked to rate their multilingual identity on a scale of 1 to 10, the participant (N16) assigned a relatively low score of 2.5, which indicates a weak or underdeveloped multilingual self. When further prompted to conceptualize their multilingual identity, the participant linked a legitimate multilingual identity explicitly to high linguistic proficiency. This interpretation reveals a belief in balanced multilingualism. This suggests that the surge in motivation does not necessarily foster a surge in multilingual awareness, and the multilingual identity is not a natural by-product of learning an additional language.

#### A subsequent reassertion of L2 motivation

As learners navigate their multilingual learning, the high motivational intensity of Japanese did not persist. The motivation for learning English regained dominance, which embodies a complex interplay between identity, capital, and value. Unsurprisingly, the strong sense of novelty derived from learning a new language soon declines as learners are confronted with increasing effort demands.

N5: After approximately eight or nine lessons in Japanese grammar, the material became particularly challenging. It seemed to disrupt my previous understanding of grammatical systems, and certain inflectional forms felt especially complex. As a result, my motivation declined.

In this case, the learning experience of increasing grammatical complexity undermined the early affective appeal. The immediate emotional reward that had initially accompanied Japanese as a fresh start diminished. More importantly, the perceived L2 fatigue was disrupted by a significant event in the institutional context, that is, the high-stakes TEM4 (Test for English Majors Band 4). This shows how high-stakes standardized tests might act as a strong, temporary factor that causes the motivational system to shift its primary orientation toward English as L2 dominance.

N4: To better prepare for the TEM-4 exam, I spent more time on English. Consequently, I am less motivated in learning Japanese.

Similar to N4, all interviewees consistently emphasized the importance of TEM4. Passing TEM4 was widely regarded as a crucial marker of becoming a qualified English major and was therefore seen as a priority that required substantial investment of time and effort. This shared perception illustrates the high-stakes exam as a symbolic threshold that legitimizes disciplinary identity, which directs resource allocation. Therefore, the motivational system shifted its dominance toward English. In complex dynamic systems terms, English regained dominance not because its intrinsic appeal increased, but because the test temporarily increased its instrumental and symbolic capital. The shift does not simply reflect a loss of interest in Japanese. However, it embodies an internalized linguistic hierarchy in which English was prioritized when conflicts arose between English and Japanese over time and resource allocation. As N15 mentioned:

N15: English is my major, and this disciplinary identity brings a certain degree of pressure to my learning. I have been exposed to the language since very young and have always aspired to master it. Consequently, the pressure associated with English is undoubtedly greater than that associated with Japanese. I approach Japanese with a relaxed and enjoyable mindset, whereas I study English with the understanding that it will constitute a professional skill and a means of livelihood in my future career. English thus takes precedence over Japanese.

Seemingly, the perceived linguistic value mediates an identity-motivation nexus. Given that English carries salient institutional, economic, and symbolic values, it is associated with identity establishment, professional legitimacy, and future employability.

Motivation then becomes the dynamic outcome of identity positioning and value attachment. English as L2 motivation is characterized by identity investment, professional development, and disciplinary obligation, whereas Japanese as L3 motivation is marked by autonomy and affection. These orientations coexist but do not equal structure. This explains the upward shift of English learning motivation toward the final phase.

N9: I feel that my role is different now. As a senior student, I find myself at a critical juncture with multiple possible pathways ahead, such as seeking employment. At this point, I realize English is a prerequisite for entering the job market.

As pointed out by N9, at the time of impending graduation, English is no longer merely an academic subject but becomes a form of convertible capital, directly linked to career access and social mobility. This identity-linked and value-laden language thus becomes the dominance. In other words, the identity-motivation nexus exerts stronger forces, which further direct motivational change toward English.

#### Asymmetrical motivational interdependence between L2 and L3

While the two themes above illustrate phase-like changes in the relative dominance of L2 and L3 motivation, the data further reveal that the relationship between English and Japanese is neither unidirectional nor symmetrical. Rather, retrospective accounts of learners suggest a pattern of asymmetrical motivational interdependence, in which the two subsystems remain interconnected yet unequal in structural strength and developmental consequences.

An important finding is that many learners perceived a limited direct influence of Japanese on their English learning motivation. As several participants explicitly stated:

N6: Learning Japanese has had no impact on my English learning.

N10: My motivation to learn English did not decline as a result of increased motivation to learn Japanese.

N14: Despite an increase in my Japanese learning motivation, I continue to study English as I did previously.

These accounts suggest that, at the level of subjective meaning-making, L2 and L3 motivation systems are often perceived to operate in parallel rather than in competition. This indicates that the English and Japanese motivational systems have developed relatively stable states. Even when one motivational system fluctuates, the overall configuration of the other motivational system is thought to remain structurally intact. However, the perceived independence does not imply systemic isolation. A closer examination of learners’ narratives reveals subtle yet meaningful cross-language influences. These experiences illustrate the integrated nature of multilingual motivation. This stands in contrast to their self-perceived understanding that their motivations for learning English and Japanese often operate in parallel. As learners reported metalinguistic influences:

N5: Memorizing new Japanese vocabulary appeared to reactivate my cognitive functions, which exerted a positive influence on my English learning.

N12: Some Japanese words are loanwords derived from English. This has benefited both my English vocabulary size and learning motivation.

As indicated in N5, the cognitive engagement involved in memorizing Japanese vocabulary appeared to enhance metalinguistic awareness and facilitate cognitive processing, which in turn exerted a beneficial effect on English learning. This highlights the cognitive benefits of multilingual learning. Meanwhile, the observation in N12 is indicative of a positive feedback loop where prior English knowledge helps Japanese learning.

While English as L2 dominates in the institutional structure, professional identity, and long-term capital, Japanese as L3 could generate both negative and positive changes. There are instances where engagement with the Japanese temporarily undermines L2 motivation.

N9: Learning Japanese occupied most of my daily time, leading me to skip some English learning activities. I believe this harmed my English learning. Later, as I needed to prepare for the TEM4. I resumed intensive English study. Subsequently, with the requirement of job hunting, English became even more important.

As the excerpt illustrates, Japanese as L3 may act as a factor that temporarily destabilizes the L2 dominance by redirecting attention and effort. English learning motivation might momentarily decline, but it did not lose its long-term dominance. Therefore, the dual system may exhibit resource-sensitive fluctuation rather than a complete phase transition. In other words, while English as L2 may repeatedly reshape the motivational landscape of L3, Japanese as L3 generally seems to exert more transient effects.

The findings indicate that English as L2 and Japanese as L3 constitute a dual motivational system characterized by asymmetrical interdependence. The subsystems are interconnected. Yet, they do not represent equivalent systemic force. Thus, the multilingual motivational system observed here cannot be reduced to simple dominance or linear suppression. Instead, it reflects a dynamic ecology in which stability and fluctuation coexist.

## Discussion

This study set out to examine how Chinese English majors retrospectively reconstruct motivational change across learning English as L2 and Japanese as L3, and how they interpret cross-language motivational dynamics within a multilingual learning trajectory. A recurrent argument in previous research is that English, due to its strong force as an international language, suppresses motivation for additional languages (Dörnyei and Al-Hoorie, 2017; Ushioda, 2017). In many Expanding Circle contexts, L3 learning is described as occurring in the shadow of English (Zeng and Yang, 2024). However, the present findings suggest that English dominance is dynamic and fluid rather than fixed.

In the early phase, Japanese temporarily surpassed English in motivational intensity. This can be interpreted as a temporary destabilization of the existing dominance hierarchy. Prior to university, English served as a stable and dominant factor by years of examination-oriented learning and prior academic success. However, the transition to university introduced a perceived discrepancy between ‘ought-to L2 learner” and ‘actual L2 learner’. The Standard Language Ideology, together with the intricate intertwining of the ideal English self and professional identity, rendered this discrepancy accompanied by a strong sense of fatigue. While discrepancies between the actual and ideal L2 self are often viewed as motivating forces (Dörnyei, 2009), recent research on language learning emotions suggests that this effect depends on a perceived sense of autonomy (Dewaele and Li, 2021). In the present study, when the perceived importance of acquiring Standard English confronts a growing awareness of insufficiency, negative emotions arise. Meanwhile, Japanese entered the system as a novel and positively framed ought-to language. In line with Henry and Liu (2024), the study found that the choice of a particular third language is often imbued with motivational aspiration. The affective contrast between L2 fatigue and L3 novelty allows a temporary shift toward L3. This corroborates that positive emotions play a crucial role in maintaining motivation in multilingual learning (Liu, 2022).

Previous research has consistently emphasized that language learning motivation is deeply intertwined with identity construction (Norton, 2013; Ushioda, 2011). However, what remains underexplored is how the issue of identity may differentially stabilize motivational systems across languages. The present findings suggest that English and Japanese were not simply associated with different future selves. Rather, they were embedded in identity projects of unequal structural consequence. English was closely tied to professional legitimacy, employability, and institutional recognition, whereas Japanese was associated more strongly to aspiration, interest, and esthetic pleasure. This asymmetry meant that the two motivational systems did not possess equivalent inertia.

In this sense, identity constituted a source of systemic stability, which conditions the resilience and persistence of one motivational subsystem over another. This explains why English learning motivation reasserted its dominance as the high-stakes TEM-4 examination drew nearer and as students progressed toward graduation. This shift does not indicate a decline in Japanese motivation per se. Instead, it brings forward the fact English tends to assume priority when the two languages compete for limited learning resources. Obviously, the competition does not occur on equal terms. Languages compete unevenly for investment, and priority tends to be allocated to the language carrying greater perceived value (Busse, 2017). In such circumstance, English was endowed greater inertia within the motivational system due to its attached value in identity formation and employability development. Thus, the motivational relationship between English and other additional languages moves beyond a simple binary of suppression or enhancement. Motivational intensity, in this sense, is not persistent but structurally conditioned.

This finding explains that identity investments may not be equally distributed across a learner’s whole linguistic repertoire, which could shape differentiated stability within multilingual motivational system. From a dynamic perspective, this implies that the strength may be partly determined by the projective role of identity associated with a particular language. A language embedded in long-term identity trajectories might accumulate more symbolic capital and becomes less vulnerable to change. Thus, such an identity-motivation nexus reconceptualizes identity as asymmetrically weighted within an interconnected motivational system, not as additive or parallel.

Interestingly, learners portrayed their English and Japanese motivational systems as independent and parallel, yet simultaneously provided examples illustrating the ways in which the two systems influenced one another. The apparent contradiction between learners’ metacognitive interpretation and the narrative of lived experience suggest a perceived-enacted motivational divergence. One possible reason is that English and Japanese are taught by different practitioners, assessed through separate evaluation schemes, and instructed in distinct pedagogies. This can be attributed “both to the ongoing maintenance of established disciplinary boundaries and the determination of what counts as disciplinary orthodoxy” (May, 2019, p. 123). The normative nature of monolingualism can reinforce what Cummins (2007) describes as the “two solitudes” assumption, whereby languages are treated as discrete systems rather than interacting resources. Under these conditions, it is unsurprising that learners internalize a view of the two motivational systems as parallel and independent. This indicates that cross-language motivational interaction may be enacted without being fully recognized at the level of meta-awareness. Seemingly, without necessary metalinguistic knowledge and multilingual awareness as emphasized by Jessner (2008), learners may fail to interpret and conceptualize what they have experienced in the multilingual trajectory. The failure, in turn, is situated within and expanded upon monolingualism, which exacerbates monolingual assumptions.

## Conclusion

This study set out to explore how Chinese English majors retrospectively reconstruct motivational change across learning English as L2 and learning Japanese as L3, and unravel how they interpret cross-language motivational dynamics within their multilingual trajectories. The findings carry several implications for multilingual education policy and practice.

The findings suggest that English learning motivational systems and Japanese learning motivational systems are interrelated systems that are simultaneously constituted within a higher-level multilingual motivational system (Henry, 2017). This motivational system is inseparable from multilingual identity development. Given that language teaching is mostly based on monolingual instruction, learning Japanese as L3 has not yet disrupted English as a legitimate identity marker and even generated a perceived sense of resource competition. Therefore, Japanese is not as equally stable as English. As such, we advocate for a translanguaging pedagogy whereby teachers of different languages can collaborate to design a curriculum that respects, activates, develops learners’ whole linguistic repertoire. As suggested by Henry (2017, p. 559), “a cross-language motivational program based on visualization techniques designed to nurture and develop multilingual identities could provide an important space where teachers can work to construct school-wide pedagogies for operating between languages.”

The findings also indicate the crucial role of emotion in regulating motivational relationships and change. As learners navigate their multilingual learning, they experience a feeling of fatigue due to failure to acquire the Standard English, a sense of novelty immediately emerging from learning a new language and gradually faded over time, a feeling of surprise upon discovering the cognitive benefits of learning two languages, and a sense of tension and anxiety in the trajectory to approximate a good language learner who is deemed capable to excel in the high-stakes examinations, confident in developing a disciplinary identity, and competitive in the job market. There is, however, not even the slightest trace of pride. Given its importance in sustaining the motivational system (MacIntyre et al., 2020), it is essential to design classroom practice with an aim to enhance the sense of pride. Teachers may consider to help learners to recognize and claim their whole linguistic repertoire, especially when learners assume languages in their repertoire “are not sufficient to qualify themselves as multilingual” (Sulis et al., 2025).

Furthermore, the perceived-enacted motivational divergence necessitates a critical understanding of learning to be a multilingual. Seemingly, university language learners, who are the weirdest multilinguals in the words of Ortega (2019), lack the abilities that grassroot multilinguals may have, to understand, interpret and conceptualize their experience in multilingual learning, classroom, and experience. If multilingual learning is to be forwarded effectively, if at all, it requires, in our view, further pedagogical thought and action in relation to metalinguistic knowledge. This could enable an appropriate meaning-making of who they are, what they have experienced and how to navigate their multilingual learning.

## Data Availability

The raw data supporting the conclusions of this article will be made available by the authors, without undue reservation.

## Appendix A: Pre-designed interview questions

Part I: Personal background

1. Could you tell me about your overall experience with learning foreign languages?
2. How do you envision your language learning and using in future?

Part II: English learning (L2)

3. Can you recall what motivated you to major in English at the beginning?
4. How would you describe your current experience of learning English?
5. When you think about your progress in English, what factors do you believe have contributed to your successes or difficulties?
6. What goals do you currently have for your English learning?
7. How do you usually plan or regulate your English learning to achieve these goals?

Part III: Japanese learning (L3)

8. What led you to choose Japanese as your third language?
9. How has your motivation to learn Japanese evolved since you started?
10. Can you describe any particular moments when your motivation for learning Japanese changed noticeably? What happened?
11. What goals do you have for your Japanese learning at this stage?
12. How do you approach planning and sustaining your Japanese learning?

Part IV: Retrospection and cross-linguistic interaction

13. Looking back, how would you describe the overall trajectory of your motivation for learning English?
14. Similarly, how has your motivation for learning Japanese changed over time?
15. How do you perceive the relationship between your English (L2) and Japanese (L3) learning experiences?
16. In what ways, if any, have the two languages influenced each other in terms of motivation, confidence, or learning strategies?
